# A Gibbs Sampler for the (Extended) Marginal Rasch Model

**DOI:** 10.1007/s11336-015-9479-4

**Published:** 2015-10-22

**Authors:** Gunter Maris, Timo Bechger, Ernesto San Martin

**Affiliations:** Cito - University of Amsterdam, Arnhem, The Netherlands; Cito, Arnhem, The Netherlands; Pontificia Universidad Catolica de Chile, Santiago, Chile

**Keywords:** item response theory, marginal Rasch model, extended Rasch model, Gibbs sampler

## Abstract

In their seminal work on characterizing the manifest probabilities of latent trait models, Cressie and Holland give a theoretically important characterization of the marginal Rasch model. Because their representation of the marginal Rasch model does not involve any latent trait, nor any specific distribution of a latent trait, it opens up the possibility for constructing a Markov chain - Monte Carlo method for Bayesian inference for the marginal Rasch model that does not rely on data augmentation. Such an approach would be highly efficient as its computational cost does not depend on the number of respondents, which makes it suitable for large-scale educational measurement. In this paper, such an approach will be developed and its operating characteristics illustrated with simulated data.

## Introduction

Over the last two decades, Markov chain - Monte Carlo (MCMC) approaches to Bayesian inference for item response theory (IRT) models have become increasingly popular. Most applications follow the data augmentation Gibbs (DA-Gibbs) approach of Albert (1992) (see also, Albert & Chib, 1993) for the normal ogive model. The work of Albert (1992) has been extended in many directions, see for instance Maris and Maris (2002), Fox and Glas (2001), Béguin and Glas (2001), and many others.

Data augmentation provides a very powerful tool to simplify sampling from distributions that are otherwise intractable. However, the tractability comes at a prize in terms of both the autocorrelation and the computational cost of every step in the resulting Markov chain, which limits its usefulness for large-scale applications.

The approach advocated by Albert (1992) involves two layers of augmented data. First, for every person an unobserved ability is introduced, and second, for every item response a normally distributed variable is introduced. Johnson and Junker (2003) propose to use a Metropolis-within-Gibbs algorithm to remove one layer of augmented data from the problem.

In this paper, a different approach will be developed that does not use data augmentation at all, and hence will give a Markov chain with lower autocorrelation, whilst at the same time producing tractable full conditional distributions. Moreover, as will become apparent later on, the computational cost for every iteration of the algorithm is independent of the number of persons. This combination makes our algorithm suitable for large-scale applications involving both large numbers of items and persons.

We take as our starting point the theoretically important characterization of the marginal Rasch model from Cressie and Holland (1983). They not only give a representation of the marginal Rasch model, but also show that without further parametric assumptions on the distribution of ability only a limited number of characteristics of the ability distribution can be estimated. Using the famous *Dutch identity* (Holland, 1990), we develop a parametrization of the marginal Rasch model in terms of item difficulty parameters, and Expected A Posteriori (EAP) estimators for ability. That is, even though the individual ability parameters do *not* figure in the Cressie and Holland (1983) characterization of the marginal Rasch model, their EAP estimators *do* figure in the model.

Recent work on the Cressie and Holland (1983) characterization of the marginal Rasch model has centred on constrained versions (Hessen, 2011; 2012), and on pseudo-likelihood approaches to parameter estimation (Anderson, Li, & Vermunt, 2007). Our work is complementary to such recent work, in that it provides researchers with a fully Bayesian approach to statistical inference suitable for use in large-scale educational measurement contexts.

This paper is organized as follows. In Sect. 2 the characterization of the marginal Rasch model from Cressie and Holland (1983) is revisited. In Sect. 3 a Gibbs sampler for the Cressie and Holland (1983) formulation of the marginal Rasch model is proposed. Section 4 provides some simulation studies to illustrate the working characteristics of our approach. Section 5 shows how the approach can be extended in a number of directions, and the paper ends with some concluding comments and discussion.

## The (Extended) Marginal Rasch Model

If *f* denotes the density for the ability distribution, the marginal Rasch model may be expressed as follows^1^:

1 $$\begin{aligned} P(\mathbf {X}=\mathbf {x}) = \int _{-\infty }^{\infty } \prod _i \frac{\exp (x_i(\theta -\delta _i))}{1+\exp (\theta -\delta _i)} f(\theta ) \mathrm{d}\theta \end{aligned}$$

where $x_i$ equals one for correct and zero for incorrect responses, $\delta _i$ is the difficulty of item *i*, and $\theta$ denotes ability.

Recognizing that

$$\begin{aligned} \frac{1}{\prod _i 1+\exp (\theta -\delta _i)} f(\theta ) \propto f(\theta |\mathbf {X}=\mathbf {0}) \end{aligned}$$

is proportional to the posterior distribution of ability for someone who answers all items incorrectly, with as proportionality constant the (marginal) probability to answer all items incorrectly ($P(\mathbf {0})$), we may express the marginal Rasch model as follows:

2 $$\begin{aligned} P(\mathbf {x})= & {} \int _{-\infty }^{\infty } \exp \left( \sum _i x_i(\theta -\delta _i)\right) f(\theta |\mathbf {X}=\mathbf {0}) \mathrm{d}\theta P(\mathbf {0}) \nonumber \\= & {} \left( \prod _i b_i^{x_i}\right) \mathcal {E}\left( \exp (x_+ \Theta )|\mathbf {X}=\mathbf {0} \right) P(\mathbf {0}) \end{aligned}$$

where $b_i=\exp (-\delta _i)$ and $x_+$ denotes the sum score.

The theoretical significance of Eq. 2, which corresponds to Equation 13 from Cressie and Holland (1983), is that it clearly shows that one cannot infer the full population distribution from the marginal Rasch model. However, theoretically, important this result is, we will treat Eq. 2 as a characterization of the marginal Rasch model that is useful for constructing a Gibbs sampler for Bayesian inference.

With some further change of notation

$$\begin{aligned} \mu= & {} P(\mathbf {0}) \\ \lambda _s= & {} \mathcal {E}\left( \exp (s \Theta )|\mathbf {X}=\mathbf {0} \right) \end{aligned}$$

we finally obtain the following characterization of the marginal Rasch model:

3 $$\begin{aligned} P(\mathbf {x}) = \prod _i b_i^{x_i} \lambda _{x_+} \mu \end{aligned}$$

As it stands, the marginal Rasch model, as written in Eq. 3, need, without further constraints, not even represent a probability distribution. A constraint which suffices to ensure that Eq. 3 represents a probability distribution (i.e. $\sum _{\mathbf {x}} P(\mathbf {x})=1$) is the following:

4 $$\begin{aligned} \mu= & {} \frac{1}{\sum _{\mathbf {x}} \prod _i b_i^{x_i} \lambda _{x_+} } \nonumber \\= & {} \frac{1}{\sum _{s =0}^{n} \gamma _s(\mathbf {b}) \lambda _s} \end{aligned}$$

in which the $\gamma _s$ function denotes the elementary symmetric function^2^ of order *s* of the vector $\mathbf {b}$.

Imposing the constraint in Eq. 4 we obtain the following expression for the marginal Rasch model

5 $$\begin{aligned} P(\mathbf {x})=p(\mathbf {x}|\mathbf {b},\varvec{\lambda }) = \frac{\prod _i b_i^{x_i} \lambda _{x_+}}{\sum _{s =0}^{n} \gamma _s(\mathbf {b}) \lambda _s} \end{aligned}$$

from which we readily see that it does indeed represent a probability distribution for all (non-negative) values of its parameters.

Additional insight in the structure of the marginal Rasch model derives from considering some of its properties. We focus on properties that are not only theoretically but also practically significant. First, from the distribution in Eq. 5 we readily find the following factorization

6 $$\begin{aligned} p(\mathbf {x}|\mathbf {b},\varvec{\lambda })= & {} p(\mathbf {x}|x_+,\mathbf {b}) p(x_+|\mathbf {b},\varvec{\lambda }) \nonumber \\= & {} \frac{\prod _i b_i^{x_i}}{\gamma _{x_+}(\mathbf {b})} \frac{\gamma _{x_+}(\mathbf {b}) \lambda _{x_+}}{\sum _{s =0}^{n} \gamma _s(\mathbf {b}) \lambda _s} \nonumber \\= & {} \frac{\prod _i b_i^{x_i}}{\gamma _{x_+}(\mathbf {b})} \pi _{x_+} \end{aligned}$$

which gives the conditional likelihood distribution that is also used in conditional maximum-likelihood estimation for the Rasch model (Andersen, 1973) and the score distribution. Observe that the factorization shows that the observed score distribution is the sufficient statistic for $\varvec{\lambda }$. Observe that the parameters $\mathbf {b}$, $\varvec{\lambda }$ and $\mathbf {b}$, $\varvec{\pi }$ are one–one transformations of each other. The last expression is due to Tjur (1982), and is called the *extended* Rasch model by Cressie and Holland (1983). We see directly from Eq. 6 that the conditional maximum- likelihood estimates of the item difficulty parameters (Andersen, 1973) are equivalent to their maximum-likelihood estimates under an extended Rasch model. As a consequence, Bayesian inferences for the parameters of the extended Rasch model can be perceived as the Bayesian analogue of conditional maximum-likelihood estimation.

Second, we consider the marginal and conditional distributions corresponding to Eq. 5. In particular, we consider the distribution of $\mathbf {x}$ without item *n* (which we denote by $\mathbf {x}^{(n)}$):

7 $$\begin{aligned} p(\mathbf {x}^{(n)}|\mathbf {b},\varvec{\lambda })= & {} p(\mathbf {x}^{(n)},1|\mathbf {b},\varvec{\lambda }) + p(\mathbf {x}^{(n)},0|\mathbf {b},\varvec{\lambda }) \nonumber \\= & {} \frac{\prod _{i\ne n} b_i^{x_i} (\lambda _{x_+^{(n)}}+\lambda _{x_+^{(n)}+1}b_n)}{\sum _{s =0}^{n} \gamma _s(\mathbf {b}) \lambda _s} \nonumber \\= & {} \frac{\prod _{i\ne n} b_i^{x_i} (\lambda _{x_+^{(n)}}+\lambda _{x_+^{(n)}+1}b_n)}{\sum _{s =0}^{n-1} \gamma _s(\mathbf {b}^{(n)}) (\lambda _s + \lambda _{s+1} b_n)} \end{aligned}$$

where the last equality follows from the following recursive property of elementary symmetric functions (Verhelst, Glas, & van der Sluis, 1984):

8 $$\begin{aligned} \gamma _s(\mathbf {b}) = \gamma _s(\mathbf {b}^{(i)})+\gamma _{s-1}(\mathbf {b}^{(i)}) b_i \end{aligned}$$

and shows that $\mathbf {X}^{(n)}$ is also a marginal Rasch model.

We readily obtain the distribution of $X_n$ conditionally on the remaining $n-1$ responses:

9 $$\begin{aligned} p(X_n=x|\mathbf {x}^{(n)},\mathbf {b},\varvec{\lambda })= & {} \frac{b_n^x \lambda _{x_+^{(n)}+x}}{\eta _{x_+^{(n)}}} = \frac{\left( b_n \frac{\lambda _{x_+^{(n)}+1}}{\lambda _{x_+^{(n)}}}\right) ^x}{1+b_n \frac{\lambda _{x_+^{(n)}+1}}{\lambda _{x_+^{(n)}}}} \nonumber \\= & {} p(X_n=x|x_+^{(n)},\mathbf {b},\varvec{\lambda }). \end{aligned}$$

We find that this conditional distribution only depends on the remaining $n-1$ responses via the raw score $x_+^{(n)}$, and it is independent of the values of the remaining item parameters $\mathbf {b}^{(n)}$. That is, expression 9 gives an analytical expression for the item-rest regression function, which may be used for evaluating the fit of the marginal Rasch model.

Third, in rewriting Eq. 2 as Eq. 3, we actually did more than just change the parametrization. Specifically, the model in Eq. 3 reduces to the model in Eq. 2 if, and only if, the $\lambda _s$ parameters represent a sequence of moments. To appreciate the kind of constraints this implies, we consider $\lambda _1$ and $\lambda _2$. From the fact that the variance of a random variable is non-negative, we readily obtain that

$$\begin{aligned} \lambda _2 = \mathcal {E}\left( \exp (2 \Theta )|\mathbf {X}=\mathbf {0} \right) \ge \mathcal {E}\left( \exp (\Theta )|\mathbf {X}=\mathbf {0} \right) ^2 = \lambda _1^2 \end{aligned}$$

In its most general form, these inequality constraints can be formulated as follows (Shohat & Tamarkin, 1943):andAfter introducing a Gibbs sampler for the extended Rasch model in Eq. 3, in the next Section, we consider how the additional constraints implied by the marginal Rasch model in Eq. 2 can be incorporated in the algorithm. In a more restricted setting, Theorem 3 of Hessen (2011) provides the constraints needed for the extended Rasch model to be equivalent to a marginal Rasch model in which the latent variable is normally distributed.

Fourth, even if all the moment constraints are met, the $\lambda _s$ parameters are not very easy to interpret, as they correspond to a sequence of moments corresponding to the posterior distribution of ability for a person who fails all the items. For that reason we introduce a more natural parametrization. Specifically, from the Dutch identity (Holland, 1990) applied to the marginal Rasch model, we immediately obtain

10 $$\begin{aligned} \tau _s= & {} \frac{\lambda _{s+1}}{\lambda _s} = \frac{\mathcal {E}(\exp ((s+1)\Theta )|\mathbf {X}=\mathbf {0})}{\mathcal {E}(\exp ((s)\Theta )|\mathbf {X}=\mathbf {0})} \nonumber \\= & {} \frac{\int _{-\infty }^{\infty } \exp ((s+1) \theta ) f(\theta |\mathbf {X}=\mathbf {0})\mathrm{d}\theta }{\int _{-\infty }^{\infty } \exp (s \theta ) f(\theta |\mathbf {X}=\mathbf {0})\mathrm{d}\theta } \nonumber \\= & {} \int _{-\infty }^{\infty } \exp (\theta ) \frac{ \frac{\exp (s \theta )}{\prod _i 1+\exp (\theta -\delta _i)} f(\theta )}{\int _{-\infty }^{\infty } \frac{\exp (s \theta )}{\prod _i 1+\exp (\theta -\delta _i)} f(\theta )\mathrm{d}\theta }\mathrm{d}\theta \nonumber \\= & {} \int _{-\infty }^{\infty } \exp (\theta ) f(\theta |X_+=s)\mathrm{d}\theta =\mathcal {E}(\exp (\Theta )| X_+=s) \end{aligned}$$

which is recognized as the posterior expectation of ability for different scores. Observe that the posterior expectation of ability for a person who answers *all* questions correctly *cannot* be estimated. As we find later, this new parametrization is also useful when considering the moment constraints implied by the marginal Rasch model. In terms of the item parameters $\mathbf {b}$ and the EAP parameters $\varvec{\tau }$, the marginal Rasch model can be expressed as follows:

$$\begin{aligned} P(\mathbf {X}=\mathbf {x}|\mathbf {b},\varvec{\tau }) = \frac{\prod _i b_i^{x_i} \prod _{s<x_+} \tau _s}{\sum _s \gamma _s(\mathbf {b}) \prod _{t<s} \tau _t} \end{aligned}$$

Fifth, a further consequence of the Dutch identity is that we can obtain not only the EAP estimators for ability, but also more generally

11 $$\begin{aligned} \frac{\lambda _{s+t}}{\lambda _s} = \mathcal {E}\left( \exp (t \Theta )|X_+=s \right) \quad , 0 \le s+t \le n \end{aligned}$$

We proceed to show how this fact can be used in combination with an algorithm to sample from the posterior distribution of the parameters of the marginal Rasch model to obtain estimates of both the posterior mean and variance of ability, taking into account the uncertainty regarding the parameters of the marginal Rasch model. Using Eq. 11, we obtain that

$$\begin{aligned} \mathcal {E}\big (\exp (\Theta )|X_+=s,\mathbf {b},\varvec{\lambda }\big ) = \frac{\lambda _{s+1}}{\lambda _s} \quad , s=0,\ldots ,n-1 \end{aligned}$$

and

$$\begin{aligned} \mathcal {E}\big (\exp (\Theta )^2|X_+=s,\mathbf {b},\varvec{\lambda }\big ) = \frac{\lambda _{s+2}}{\lambda _s} \quad , s=0,\dots ,n-2 \end{aligned}$$

from which we directly obtain (for $s=0,\ldots ,n-1$)

$$\begin{aligned} \mathcal {E}\big (\exp (\Theta )|X_+=s,\mathbf {X}=\mathbf {x}\big )= & {} \mathcal {E}[\mathcal {E}(\exp (\Theta )|X_+=s,\mathbf {B},\varvec{\Lambda })|\mathbf {X}=\mathbf {x}] \\= & {} \mathcal {E}\left[ \frac{\Lambda _{s+1}}{\Lambda _s}|\mathbf {X}=\mathbf {x}\right] \end{aligned}$$

which can be directly estimated (using Monte Carlo integration) with a sample from the posterior distribution of $\varvec{\Lambda }$. Similarly, we can estimate the posterior variance of ability (for $s=0,\dots ,n-2$)

12 $$\begin{aligned} \mathcal {V}(\exp (\Theta )|X_+=s,\mathbf {X}=\mathbf {x})= & {} \mathcal {V}[\mathcal {E}(\exp (\Theta )|X_+=s,\mathbf {B},\varvec{\Lambda })|\mathbf {X}=\mathbf {x}] \nonumber \\&+\, \mathcal {E}[\mathcal {V}(\exp (\Theta )|X_+=s,\mathbf {B},\varvec{\Lambda })|\mathbf {X}=\mathbf {x}] \end{aligned}$$

where $\mathcal {V}(\exp (\Theta )|X_+=s,\mathbf {b},\varvec{\lambda })$ is estimated as follows:

$$\begin{aligned} \mathcal {V}(\exp (\Theta )|X_+=s,\mathbf {b},\varvec{\lambda }) = \frac{\lambda _{s+2}}{\lambda _s} - \left( \frac{\lambda _{s+1}}{\lambda _s}\right) ^2 \end{aligned}$$

The first term on the right-hand side of Eq. 12 reflects uncertainty due to the fact that the parameters $\mathbf {b}$ and $\varvec{\lambda }$ are not known, whereas the second term reflects uncertainty due to finite test length. Specifically, as the number of persons tends to infinity, the first term in Eq. 12 tends to zero. The second term, however, tends to zero as the number of items tends to infinity.

For some, inferences regarding $\exp (\theta )$ rather than regarding $\theta$ directly may seem inconvenient. Particularly, since the posterior distribution of $\exp (\theta )$ converges to its asymptotic (in the number of items) normal limit at a slower rate than does the posterior distribution of $\theta$. Hence, the posterior mean and variance of $\exp (\theta )$ need not give a good summary of the posterior distribution. Using Corollary 1 from Holland (1990), we may for scores for which the posterior distribution of $\theta$ can be considered to be approximately normal, use the relation between moments of the log-normal distribution, and the mean and variance of the corresponding normal distribution to obtain approximations to the posterior mean and variances of $\theta$ (denoted below with $\mu _s$ and $\sigma ^2_s$):

$$\begin{aligned} \mathcal {E}(\exp (\Theta )|X_+=s,\mathbf {b},\varvec{\lambda })\approx \exp (\mu _s + 1/2 \sigma ^2_s) \end{aligned}$$

and

$$\begin{aligned} \mathcal {E}(\exp (\Theta )^2|X_+=s,\mathbf {b},\varvec{\lambda })\approx \exp (2\mu _s + 2 \sigma ^2_s) \end{aligned}$$

such that

$$\begin{aligned} \sigma ^2_s \approx \ln [\mathcal {E}(\exp (\Theta )^2|X_+=s,\mathbf {b},\varvec{\lambda })] - 2 \ln [\mathcal {E}(\exp (\Theta )|X_+=s,\mathbf {b},\varvec{\lambda })] \end{aligned}$$

and

$$\begin{aligned} \mu _s \approx 2 \ln [\mathcal {E}(\exp (\Theta )|X_+=s,\mathbf {b},\varvec{\lambda })] - \ln [\mathcal {E}(\exp (\Theta )^2|X_+=s,\mathbf {b},\varvec{\lambda })]/2 \end{aligned}$$

Finally, in the field of educational surveys (such as PISA, TIMMS, ESLC, etc.), the purpose of the study is to relate ability to student (or school, or system) characteristics. We shortly consider how such research could, in principle, be based on the marginal Rasch model. In typical applications, the relation between student responses and other student characteristics (e.g. gender) runs through ability. That is $\mathbf {Y}$ (the student characteristics) and the student responses $\mathbf {X}$ are independent conditionally on ability. Typically, the distribution of ability conditionally on $\mathbf {Y}$ is modelled as a normal regression model.

### **Theorem 1**

If  and , then also 

### *Proof*

The conditions of the Theorem imply the following joint distribution:

$$\begin{aligned} f(\mathbf {x},x_+,\mathbf {y},\theta ) = f(\mathbf {y}|\theta ) p(\mathbf {x}|x_+) p(x_+|\theta ) f(\theta ) \end{aligned}$$

from which we immediately obtain

$$\begin{aligned} f(\mathbf {y},\mathbf {x}|x_+) = p(\mathbf {x}|x_+) \int _{-\infty }^{\infty } f(\mathbf {y}|\theta ) f(\theta |x_+) \mathrm{d}\theta \end{aligned}$$

$$\square $$

Theorem 1 shows that under the assumptions of independence between $\mathbf {X}$ and $\mathbf {Y}$ conditionally on $\theta$, and of sufficiency of the sum score, all information on the relation between $\mathbf {Y}$ and $\mathbf {X}$ is contained in the distribution of $\mathbf {Y}$ conditionally on $X_+$, which is (at least in principle) directly observable (to any desired degree of accuracy). Observe that Theorem 1 holds true for every element of $\mathbf {Y}$ in isolation, which implies that we may model *main effects* of student characteristics with an appropriate item-rest regression function (with the item relating to an element of $\mathbf {Y}$, and the rest to $X_+$). Observe furthermore that, using Bayes theorem, we may equally well estimate the distribution of $X_+$ conditionally on an element from $\mathbf {Y}$.

## A Gibbs Sampler

Looking at the likelihood function in Eq. 5, we readily see that the parameters are not identifiable from $\mathbf {X}$. Specifically, using the following well-known relation for elementary symmetric functions $\gamma _s(c \mathbf {b})=c^s \gamma _s(\mathbf {b})$ (Verhelst et al., 1984), we obtain

$$\begin{aligned} p(\mathbf {x}|\mathbf {b},\varvec{\lambda })= & {} \frac{\prod _i b_i^{x_i} \lambda _{x_+}}{\sum _{s =0}^{n} \gamma _s(\mathbf {b}) \lambda _s} \\= & {} \frac{\prod _i b_i^{x_i} \frac{c^{x_+}}{c^{x_+}}\lambda _{x_+}}{\sum _{s =0}^{n} \gamma _s(\mathbf {b}) \frac{c^s}{c^s} \lambda _s} \\= & {} \frac{\prod _i (cb_i)^{x_i} \lambda ^*_{x_+}}{\sum _{s =0}^{n} \gamma _s(c\mathbf {b}) \lambda ^*_s} = p(\mathbf {x}|c\mathbf {b},\varvec{\lambda }^*) \end{aligned}$$

with $\lambda ^*_s=\lambda _s/c^s$. This type of non-identifiability can easily be resolved with a constraint on one of the item parameters $b_i=1$ (which we assume to be the first one, without loss of generality). Observe, however, that changing the identifying constraint also changes the values of $\varvec{\lambda }$. Observe, that the $\lambda _s$ may all be multiplied with the same constant, without changing the distribution. This additional type of non-identifiability can easily be resolved by constraining one of the $\lambda _s$ parameters to a constant.

In order to construct an algorithm for sampling from the posterior distribution of $\mathbf {b}$ and $\varvec{\lambda }$ corresponding to Eq. 5, a prior distribution needs to specified. We consider a simple prior distribution for the parameters which give rise to tractable full conditional distributions for each of the parameters. The prior we consider is the following:

13 $$\begin{aligned} f(\mathbf {b},\varvec{\lambda }) = \left( \prod _i \alpha _i b_i^{\alpha _i-1}\right) \left( \prod _s \beta _s \lambda _s^{\beta _s-1}\right) \end{aligned}$$

Assuming that none of the items is answered (in)correctly by all students, and that every score occurs at least once, we can specify an improper uniform prior distribution of $\mathbf {b}$ and $\varvec{\lambda }$ by choosing all $\alpha _i$ and $\beta _s$ to be equal to one:

$$\begin{aligned} f(\mathbf {b},\varvec{\lambda })\propto 1 \end{aligned}$$

that still yields a proper posterior distribution.

Using this prior, the posterior distribution is the following:

14 $$\begin{aligned} f(\mathbf {b},\varvec{\lambda }|\mathbf {x};\varvec{\alpha },\varvec{\beta }) \propto \frac{\prod _i b_i^{x_{+i}+\alpha _i-1} \prod _s \lambda _s^{m_s+\beta _s-1}}{\left( \sum _{s =0}^{n} \gamma _s(\mathbf {b}) \lambda _s\right) ^m} \end{aligned}$$

where $x_{+i}$ refers to the number of persons that make item *i* correct, $m_s$ refers to the number of persons that obtain a sum score equal to *s*, and *m* denotes the number of persons.

The distribution in Eq. 14 is not very tractable. Specifically, it is not immediately clear how to generate iid draws from it. We show that using a Gibbs sampler (Geman & Geman, 1984; Gelfand & Smith, 1990; Casella & George, 1992) we obtain full conditional distributions that are each easy to sample from. In that way, we can generate a Markov chain for which the posterior distribution in Eq. 14 is the unique invariant distribution.

### Full Conditional Distribution for $b_i$

The full conditional distribution for an item parameter $b_i$ is proportional to

15 $$\begin{aligned} f(b_i|\mathbf {b}^{(i)},\varvec{\lambda },\mathbf {x};\varvec{\alpha },\varvec{\beta }) \propto \frac{b_i^{x_{+i}+\alpha _i-1} }{\left( \sum _{s =0}^{n} \gamma _s(\mathbf {b}) \lambda _s\right) ^m} \end{aligned}$$

In order to see how a sample from the full conditional distribution in Eq. 15 may be generated, we use the recursive property of elementary symmetric functions in Eq. 8 which shows that elementary symmetric functions are linear in each of their arguments.

Using the result in Eq. 8 allows us to rewrite the full conditional distribution in Eq. 15 as follows:

16 $$\begin{aligned} f(b_i|\mathbf {b}^{(i)},\varvec{\lambda },\mathbf {x};\varvec{\alpha },\varvec{\beta }) \propto \frac{b_i^{x_{+i}+\alpha _i-1} }{\left( 1+c b_i \right) ^m} \end{aligned}$$

where *c* is a constant depending only on all *other* parameters:

$$\begin{aligned} c = \frac{\sum _{s =0}^{n} \gamma _{s-1}(\mathbf {b}^{(i)}) \lambda _s}{\sum _{s =0}^{n} \gamma _{s}(\mathbf {b}^{(i)}) \lambda _s} \end{aligned}$$

With a transformation of variables

17 $$\begin{aligned} y=\frac{c b_i}{1+c b_i} \end{aligned}$$

we obtain the following expression

18 $$\begin{aligned} f(y|\mathbf {b}^{(i)},\varvec{\lambda },\mathbf {x};\varvec{\alpha },\varvec{\beta }) \propto y^{x_{+i}+\alpha _i-1} (1-y)^{m-x_{+i}-\alpha _i-1} \end{aligned}$$

which is readily seen to be a beta distribution.

That is, if we generate *y* from a beta ($x_{+i}+\alpha _i,m-x_{+i}-\alpha _i$) distribution, then the following transformation of *y* (being the inverse to the transformation in Eq. 17)

$$\begin{aligned} b_i = \frac{1}{c} \frac{y}{1-y} \end{aligned}$$

gives us a draw from the full conditional distribution in Eq. 15. Formally, the distribution in Eq. 15 classifies as a *scaled Beta prime* distribution.

### Full Conditional Distribution for $\lambda _s$

The full conditional distribution for an element of $\varvec{\lambda }$ is readily seen to be the following:

19 $$\begin{aligned} f(\lambda _t|\mathbf {b},\varvec{\lambda }^{(t)},\mathbf {x};\varvec{\alpha },\varvec{\beta }) \propto \frac{\lambda _t^{m_t+\beta _t-1}}{\left( \sum _{s =0}^{n} \gamma _s(\mathbf {b}) \lambda _s\right) ^m} \end{aligned}$$

As we found when considering the full conditional distribution for the item parameters, we see that the denominator in Eq. 19 is linear in $\lambda _t$, such that we obtain

20 $$\begin{aligned} f(\lambda _t|\mathbf {b},\varvec{\lambda }^{(t)},\mathbf {x};\varvec{\alpha },\varvec{\beta }) \propto \frac{\lambda _t^{m_t}}{\left( 1+c \lambda _t\right) ^m} \end{aligned}$$

where now the constant (with respect to $\lambda _t$) *c* equals

$$\begin{aligned} c=\frac{\gamma _t(\mathbf {b})}{\sum _{s\ne t} \gamma _s(\mathbf {b}) \lambda _s} \end{aligned}$$

We see that the full conditional distributions for both the $b_i$ and the $\lambda _s$ parameters belong to the same family of distributions.

## Simulation Results

In this section we present some simulation results to illustrate the operating characteristics of our new Gibbs sampler. We focus on two aspects. First, we evaluate the autocorrelation in the Markov chain, which drives convergence. Second, we evaluate the computational burden. In Appendix an illustrative implementation of our Gibbs sampler is given in R (R Development Core Team, 2011). This code was used to generate the simulation results presented below. Observe that when *n* becomes large, significant computational advantages can be obtained by coding (parts of) the algorithm in a compiled language (e.g. C++, Fortran, Pascal). All simulations were run on a Lenovo X200s laptop with an Intel Core2 Duo CPU with a clock speed of 2.13 GHz and 2 gigabytes of memory running Windows 7 Enterprise.

### Autocorrelation and Convergence

Convergence of Markov chains is driven by the autocorrelation structure of the chain. In this simulation study we evaluate the autocorrelation as a function of lag, and convergence of the Gibbs sampler. A Markov chain is converged in iteration *t* if the cumulative distribution function (CDF) at iteration *t* and $t+1$ coincide. For a 30 item test, with true item difficulties uniformly distributed between $-2$ and 2, and 100,000 persons drawn from a standard normal distribution, 5000 replications of the Gibbs sampler were run for 50 iterations each, with starting values uniformly distributed between 0 and 1 for *b*, and $\lambda$. These 5000 Markov chains allow us to estimate the autocorrelation between any two iterations, and to evaluate the distribution of every parameter at every iteration.

**Fig. 1:**
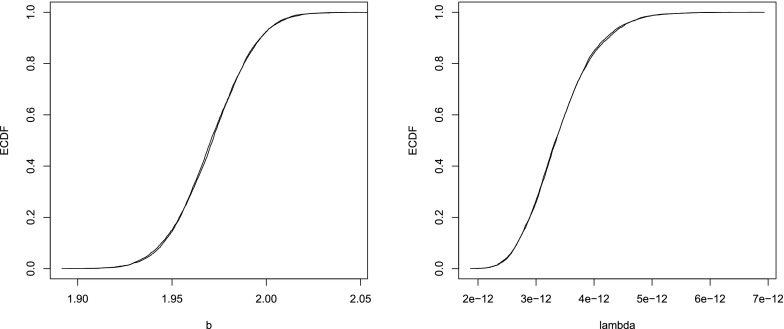
Empirical distribution functions for iterations 49 and 50 based on 5000 replications of the Gibbs sampler for $b_2$ and $\lambda _{10}$.

Figure 1 shows the empirical CDF (ECDF) after 49 and 50 iterations for one item parameter ($b_2$) and one of the $\lambda$ parameters ($\lambda _{10}$). It is clear from Figure 1 that after only 50 iterations the Markov chain is converged.

**Fig. 2:**
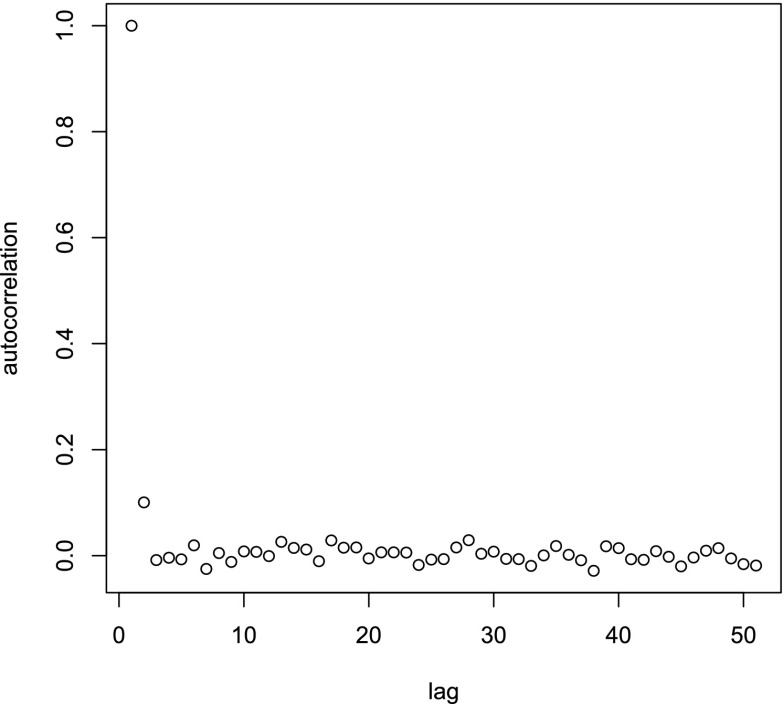
Autocorrelation for lag 0 to 50, after a burnin of 49 iterations, for $b_2$ based on 5000 replications of the Gibbs sampler.

Figure 2 gives the autocorrelation for lag 0 to 50, after discarding the first 49 iterations. It is clear from Figure 2 that except for the lag 1 autocorrelation, autocorrelation is negligible.

We conclude that our Markov chain comes close to generating an independent and identically distributed sample from the posterior distribution, with virtually no autocorrelation whatsoever.

### Computational Complexity

An algorithm for which the computational cost does not depend on the number of persons has *in principle* great advantages over algorithms for which the computational cost increases with the number of persons. For instance, we can guarantee that for some sample size $m^*$ our algorithm will outperform any particular competitor for which the computational cost increases with sample size. However, it is only practically relevant if $m^*$ is some modest number. Clearly, if $m^*$ equals $10^9$ there is little need for our algorithm. Moreover, the question remains whether our algorithm is feasible for realistic sample sizes. For instance, if for 30 items and $10^5$ persons, one iteration takes a week, our algorithm may be more feasible than competitors, but still not feasible.

**Fig. 3:**
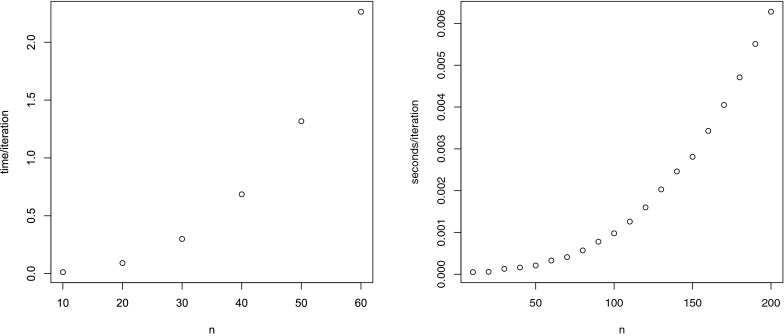
Number of items (*n*) versus average time per iteration (in seconds) for the GNU R implementation (*left panel*) and a C implementation (*right panel*).

To evaluate the feasibility of the algorithm, the average time for one iteration for tests with a different number of items, and 100,000 persons, is given in Figure 3 (left panel).

The average time per iteration appears to increase as a quadratic function of the number of items. The largest cost per iteration is in the repeated evaluation of elementary symmetric functions, the computational complexity of which is quadratic in the number of items.

To illustrate the computational gain from coding the algorithm in a compiled language, we compare the naive R implementation that is in Appendix with a C implementation of both the full conditional distribution for *b* and $\lambda$ that is called from within R using a dynamic link library. The right panel of Figure 3 gives results on the computational time per iteration for tests consisting of different numbers of items. We see that even for a test consisting of 200 items, we can do roughly 150 iterations per second, regardless of the number of students. Comparing the right with the left-hand panel in Figure 3 shows the dramatic improvement that results from implementing key parts of the algorithm in C (or Fortran, etc.).

**Fig. 4:**
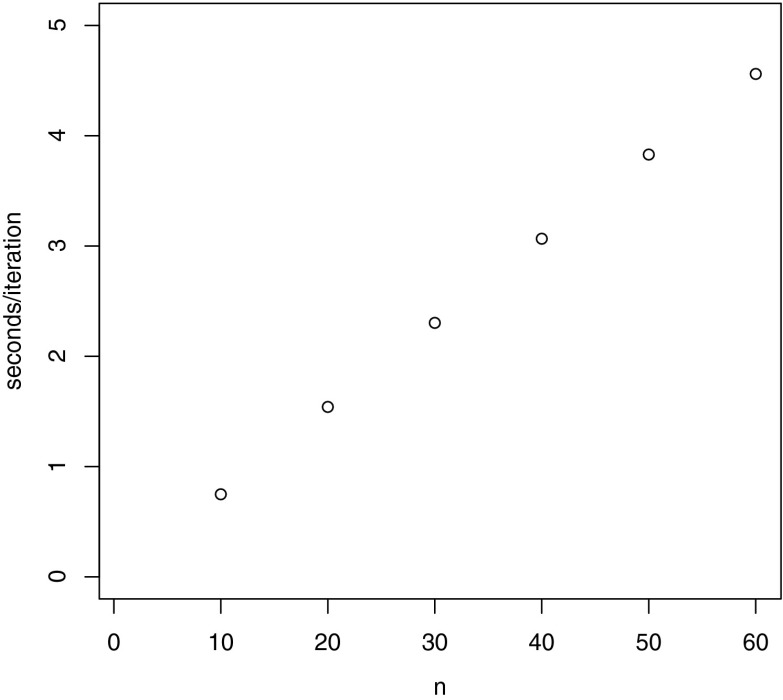
Number of items (*n*) versus average time per iteration (in seconds) for a C implementation of the DA-Gibbs sampler of Albert (1992).

Finally, for comparison, the DA-Gibbs sampler of Albert (1992), or the Metropolis-within-Gibbs sampler of Johnson and Junker (2003) have a computational cost that increases as a linear function of both the number of items and persons. For the DA-Gibbs sampler we illustrate the average time for one iteration, for a C implementation, with different numbers of items and 100,000 persons, in Figure 4. We see in Figure 4 that the average time per iteration increases as a linear function of the number of items, and is considerably larger than the average times for our new algorithm when implemented in C.

### Conclusion

The combination of low autocorrelation that implies a low number of burn in iterations to reach convergence of the Markov chain, and a small number of iterations after convergence on which inferences will be based, together with a cost per iteration that only depends on the number of items (such that for a test of 200 items we can do 9000 iterations a minute), make our Gibbs sampler extremely feasible, even for very large-scale applications.

## Extensions

The approach taken to estimate the parameters of the marginal Rasch model can easily be generalized in various directions. To illustrate its flexibility, we consider dealing with incomplete designs, dealing with polytomous responses, dealing with multidimensional Rasch models and incorporating moment constraints. As will become clear, all of these generalizations can be combined with each other without losing the desirable characteristics of the simple algorithm presented above.

### Incomplete Designs

The first problem we tackle is to show how the marginal Rasch model works out for data collected with a non-equivalent groups anchor test (NEAT) design. We consider the simplest NEAT design explicitly, but all results carry over immediately to more complicated designs.

Consider two groups of students, from possibly different populations, taking a test that consists of an anchor (we use $\mathbf {x}$ to denote responses on the anchor, and $\mathbf {b}$ for its item parameters), and a unique set of items (we use $\mathbf {y}$ and $\mathbf {z}$ for responses on the unique sets, and $\mathbf {c}$ and $\mathbf {d}$ for their parameters). Applying our representation for the marginal Rasch model we obtain the following two distributions:

$$\begin{aligned} p(\mathbf {x},\mathbf {y}) = \frac{\prod _i b_i^{x_i} \prod _j c_j^{y_j} \lambda _{x_++y_+}}{\sum _s \gamma _s(\mathbf {b},\mathbf {c}) \lambda _s} \end{aligned}$$

and

$$\begin{aligned} p(\mathbf {x},\mathbf {z}) = \frac{\prod _i b_i^{x_i} \prod _k d_k^{z_k} \eta _{x_++z_+}}{\sum _t \gamma _t(\mathbf {b},\mathbf {d}) \eta _t} \end{aligned}$$

It is immediately clear that for the parameters $\mathbf {c},\mathbf {d},\varvec{\lambda }$ and $\varvec{\eta }$, we obtain the same full conditional distributions as before. For the anchor items, the full conditional becomes the following:

$$\begin{aligned} f(b_i|\mathbf {b}^{(i)},\mathbf {c},\mathbf {d},\varvec{\lambda },\varvec{\eta },\mathbf {x},\mathbf {y},\mathbf {z};\varvec{\alpha },\varvec{\beta }) \propto \frac{b_i^{x_{+i}+\alpha _i-1}}{(\sum _s \gamma _s(\mathbf {b},\mathbf {c}) \lambda _s )^{m_{xy}} (\sum _t \gamma _t(\mathbf {b},\mathbf {d}) \eta _t)^{m_{xz}}} \end{aligned}$$

which can be rewritten to the following general form:

$$\begin{aligned} f(b_i|\mathbf {b}^{(i)},\mathbf {c},\mathbf {d},\varvec{\lambda },\varvec{\eta },\mathbf {x},\mathbf {y},\mathbf {z};\varvec{\alpha },\varvec{\beta }) \propto \frac{b_i^{x_{+i}+\alpha _i-1}}{(1+a_1 b_i)^{m_{xy}} (1+a_2 b_i)^{m_{xz}}} \end{aligned}$$

which classifies as a *rational distribution*.

With a further transformation of variables used

$$\begin{aligned} \delta _i=-\ln (b_i) \end{aligned}$$

we obtain

$$\begin{aligned} f(\delta _i|\mathbf {b}^{(i)},\mathbf {c},\mathbf {d},\varvec{\lambda },\varvec{\eta },\mathbf {x},\mathbf {y},\mathbf {z};\varvec{\alpha },\varvec{\beta }) \propto \frac{\exp (-[x_{+i}+\alpha _i]\delta _i)}{(1+a_1 \exp (-\delta _i))^{m_{xy}} (1+a_2 \exp (-\delta _i))^{m_{xz}}} \end{aligned}$$

It is readily found that the natural logarithm of this distribution is concave and has linear tails and a single mode:

21 $$\begin{aligned}&\frac{\partial }{\partial \delta _i} \ln (f(\delta _i|\mathbf {b}^{(i)},\mathbf {c},\mathbf {d},\varvec{\lambda },\varvec{\eta },\mathbf {x},\mathbf {y}, \mathbf {z};\varvec{\alpha },\varvec{\beta })) \nonumber \\&\quad \rightarrow {\left\{ \begin{array}{ll} -(x_{+i}+\alpha _i) &{} \,\, \text {as }\quad \delta _i \rightarrow \infty \\ (m_{xy}+m_{xz})-(x_{+i}+\alpha _i) &{}\,\, \text {as }\quad \delta _i \rightarrow -\infty \end{array}\right. } \end{aligned}$$

Since the distribution is log-concave, we may use the adaptive rejection sampler from Gilks and Wild (1992). As an alternative, we propose a Metropolis sampler with a proposal distribution that closely matches the full conditional distribution.

**Fig. 5:**
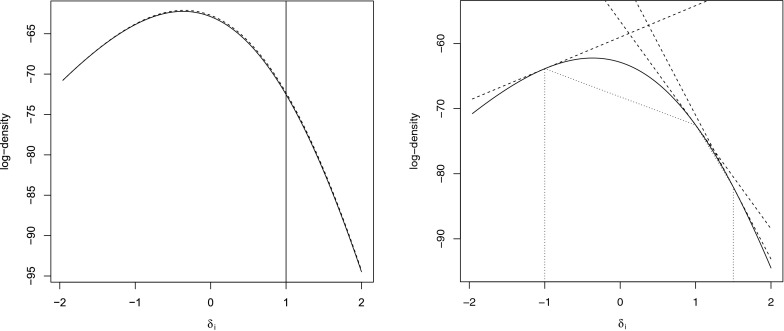
The *solid line* (in both panels) gives the log full conditional in a NEAT design. In the *left panel*, the *dashed line* gives the log of our proposal. In the *right panel*, the *dashed line* gives the *upper hull* and the *dotted line* the *lower hull* for adaptive rejection sampling density.

As a proposal distribution we consider the following distribution:

$$\begin{aligned} g(\delta _i) \propto \frac{\exp (-[x_{+i}+\alpha _i]\delta _i)}{\left( 1+c \exp (-\delta _i)\right) ^{m_{xy}+m_{xz}}} \end{aligned}$$

the logarithm of which has linear tails with the same slope, which is recognized to be of the same form as the full conditional distribution for $b_i$ found with a complete design (i.e. Eq. 16 with a transformation of variables). We propose to choose the parameter *c* in such a way that the derivative of the logarithm of the proposal distribution with respect to $\delta _i$ matches the value found for the target full conditional distribution, at its current value in the Markov chain. This proposal distribution closely matches the target full conditional distribution, as is illustrated in Figure 5 (left panel), which ensures that the resulting Metropolis-within-Gibbs algorithm will converge rapidly to its invariant distribution. For comparison, the right panel in Figure 5 gives the outer and inner hull for an adaptive rejection sampler based on three support points. Based on this comparison, we expect our Metropolis algorithm to outperform the adaptive rejection sampler, although either algorithm will work.

### Multidimensional Model

A second generalization we want to consider is a situation where we have two tests measuring different constructs administered to a group of students. That is, we consider the following marginal likelihood:

$$\begin{aligned} P(\mathbf {x},\mathbf {y}) = \int _{-\infty }^{\infty } \int _{-\infty }^{\infty } \prod _i \frac{\exp (x_i(\theta -\delta _i))}{1+\exp (\theta -\delta _i)} \prod _j \frac{\exp (y_j(\eta -\beta _i))}{1+\exp (\eta -\beta _i)} f(\theta ,\eta ) {\text {d}}\theta {\text {d}}\eta \end{aligned}$$

Using the same approach as taken for the marginal Rasch model we obtain the following representation:

$$\begin{aligned} P(\mathbf {x},\mathbf {y})= & {} \prod _i \exp (-x_i \delta _i) \prod _j \exp (-y_j \beta _j) \mathcal {E}(\exp (x_+ \Theta +y_+ H|\mathbf {X}=\mathbf {0}, \mathbf {Y}=\mathbf {0})\\&P(\mathbf {X}=\mathbf {0}, \mathbf {Y}=\mathbf {0}) \end{aligned}$$

which may be reparameterized to

$$\begin{aligned} p(\mathbf {x},\mathbf {y}|\mathbf {b},\mathbf {c},\varvec{\lambda }) = \frac{\prod _i b_i^{x_i} \prod _j c_j^{y_j} \lambda _{x_+,y_+}}{\sum _{s,t} \gamma _s(\mathbf {b}) \gamma _t(\mathbf {c}) \lambda _{s,t}} \end{aligned}$$

We readily find that all full conditionals will be of the same general form as those for the marginal Rasch model.

### Polytomous Responses

As a generalization of the Rasch model for polytomous items we consider a special case of the *Nominal Response Model* (Bock, 1972) namely one with a fixed scoring rule. The Gibbs sampler for this model will be developed along the same lines as that for the Rasch model.

Consider an item *i* with $J_i+1$ response alternatives $j=0,\dots ,J_i$; one of which is chosen. Let $X_{pi}$ denote the response alternative and for practical reasons we also consider the dummy coded variables $Y_{ij} = 1$ if category *j* was chosen and $Y_{ij}=0$ otherwise. The category response function of the NRM is given by

22 $$\begin{aligned} P(X_i=j)=P(Y_{ij} = y_{ij}| \theta ) = \frac{\exp \left[ y_{ij}(a_{ij}\theta -\delta _{ij})\right] }{\sum _{h} \exp (a_{ih}\theta -\delta _{ih})} \end{aligned}$$

where $a_{i0}=\delta _{i0}=0$ for identification. We assume that the parameters $a_{ij}$ are known integer constant and the NRM specializes to an exponential family model in which $y_{++}=\sum _i \sum _j a_{ij}y_{ij}=\sum _i a_{i,x_{pi}}$ is a sufficient statistic for $\theta _p$. Among others the *One Parameter Logistic Model* (OPLM: Verhelst & Glas, 1995) and the partial credit model (e.g. Masters, 1982) are special cases that satisfy these additional constraints.

A derivation of the Gibbs sampler for this model proceeds along the same lines as before. First, with $\prod _{i,j}$ as a shorthand notation for the product $\prod _{i}\prod ^{J_i}_{j=1}$

23 $$\begin{aligned} P(\mathbf {y})=\left( \prod _{i,j} b_{ij}^{y_{ij}}\right) \mathcal {E}\left( e^{y_{++}\Theta }|\mathbf {X}=\mathbf {0}\right) P(\mathbf {0}) \end{aligned}$$

where $b_{ij}=\exp (-\delta _{ij})$ and $X=0$ denotes a response pattern where zero credit was earned on each of the items. Thus, we obtain the following parametrization of the marginal model:

24 $$\begin{aligned} P(\mathbf {x})=\frac{\prod _{ij} b^{y_{ij}}_{ij}}{\sum _s \gamma _s(\mathbf {b})\lambda _s} \end{aligned}$$

where

25 $$\begin{aligned} \gamma _s(\mathbf {b})=\sum _{y \rightarrow s} \prod _{i,j} b_{ij}^{y_{ij}} \end{aligned}$$

are the elementary functions which satisfy the recursion

26 $$\begin{aligned} \gamma _{y_{++}}(\mathbf {b}) = \gamma _{y_{++}}(\mathbf {b}^{(i)}) + \sum ^{J_i}_{h=1} b_{ih} \gamma _{y_{++}-a_{ih}}(\mathbf {b}^{(i)}) \end{aligned}$$

Note that all formulae specialize to those for the dichotomous Rasch model when $J_i=1$ for all *i*, and $a_{ij}=1$ for $j=1$. Using the recursive property of the elementary symmetric functions, it follows that the denominator in the expression for $P(\mathbf {x})$ is linear in individual parameters which means that the Gibbs sampler for the polytomous model will be similar to the one for the Rasch model. The difference is in the normalizing constants for the full conditional distributions.

### Parameter Constraints

As observed above, the extended Rasch model reduces to the marginal Rasch model if, and only if, certain constraints on the $\lambda _s$ parameters are met. Here we consider how parameter constraints can be incorporated in the Gibbs sampler. We focus on two different types of constraints. On the one hand we consider imposing *some* of the moment constraints on the $\lambda _s$ parameters. On the other hand we show how to constrain the $\lambda _s$ parameters such that the model reproduces moments of the score distribution, rather than the complete score distribution.

Before, we found that $\lambda _2 - \lambda _1^2 \ge 0$, is one (and probably the simplest) of the moment constraints. However, all constraints are formulated as a function of a set of $\lambda _s$ parameters that needs to be non-negative. Hence, the marginal Rasch model corresponds to an extended Rasch model with particular inequality constraints on the $\lambda _s$ parameters.

In contrast to maximum-likelihood-based inference, Bayesian MCMC algorithms are particularly well suited for incorporating inequality constraints between parameters for the purpose of parameter estimation. Before illustrating this, we first recast the moment constraints in a different form, which is important for educational measurement purposes.

Using Eq. 11, we obtain from the non-negativity of the (posterior) variance (for every score) that

27 $$\begin{aligned} \frac{\lambda _{s+2}}{\lambda _s} \ge \left( \frac{\lambda _{s+1}}{\lambda _s}\right) ^2 \end{aligned}$$

which we can equivalently express as

28 $$\begin{aligned} \mathcal {E}\left( \exp (\Theta )|X_+=s+1 \right) = \frac{\lambda _{s+2}}{\lambda _{s+1}} \ge \frac{\lambda _{s+1}}{\lambda _s} =\mathcal {E}\left( \exp (\Theta )|X_+=s \right) \end{aligned}$$

This expression is important, as it implies that the $\tau$ parameters are a monotone function of the score, which is the minimal constraint on the extended Rasch model needed for educational measurement purposes.

We now consider how the Gibbs sampler can be adapted, to incorporate the inequality constraints in Eq. 28. In a Bayesian framework, inequality constraints are introduced through the prior distribution. Specifically, we obtain the following prior distribution for the $\lambda$ parameters:

$$\begin{aligned} f(\varvec{\lambda }) \propto \left( \prod _s \beta _s \lambda _s^{\beta _s-1} \right) \left( \frac{\lambda _1}{\lambda _0} \le \frac{\lambda _2}{\lambda _1} \le \frac{\lambda _3}{\lambda _2} \le \cdots \frac{\lambda _n}{\lambda _{n-1}} \right) \end{aligned}$$

With this prior distribution, the full conditional distribution for, say, $\lambda _2$ becomes

29 $$\begin{aligned} f(\lambda _2|&\mathbf {b},\varvec{\lambda }^{(2)},\mathbf {x};\varvec{\alpha },\varvec{\beta }) \nonumber \\&\propto \frac{\lambda _2^{m_2+\beta _2-1}}{\left( \sum _{s =0}^{n} \gamma _s(\mathbf {b}) \lambda _s\right) ^m} \left( \frac{\lambda _1}{\lambda _0} \le \frac{\lambda _2}{\lambda _1} \le \frac{\lambda _3}{\lambda _2} \le \frac{\lambda _4}{\lambda _3}\right) \nonumber \\&\propto \frac{\lambda _2^{m_2+\beta _2-1}}{\left( \sum _{s =0}^{n} \gamma _s(\mathbf {b}) \lambda _s\right) ^m} \left( \max (\frac{\lambda _1^2}{\lambda _0},\frac{\lambda _3^2}{\lambda _4}) \le \lambda _2 \le \sqrt{\lambda _1 \lambda _3} \right) \end{aligned}$$

We find that all that is needed is an algorithm for sampling from a double-truncated scaled Beta prime distribution, which is a fully tractable problem.

The extended Rasch model is an exponential family model with as sufficient statistics the observed number of students answering each item correct, and the observed score distribution. If we impose a log-polynomial constraint on the $\lambda _s$ parameters:

$$\begin{aligned} \log \lambda _s = \sum _{j=0}^{J} \alpha _j s^j \end{aligned}$$

we effectively replace the entire score distribution as sufficient statistics with the first *J* non-central moments of the score distribution. This effectively smooths the observed score distribution.

## Discussion

The algorithm proposed in this paper provides a flexible, robust, and highly efficient approach to Bayesian inference for the marginal Rasch model.

As opposed to maximum-likelihood estimation, our Bayesian approach (a) allows for accounting for all sources of uncertainty in the model parameters (especially in the posterior expectation of ability), (b) does not need computation and inversion of the information matrix (both of which are computationally expensive) and (c) allows for imposing moment constraints. This last point allows for considering models that are more restrictive than the extended Rasch model, yet less restrictive than the typical marginal Rasch model (i.e. assuming a normal distribution for ability).

The various generalizations we considered (incomplete data, polytomous responses, multidimensional marginal Rasch models, moment constraints) demonstrate the flexibility of our approach. The efficiency of our approach derives from the fact that no form of data augmentation is used. This not only is highly beneficial in terms of the resulting autocorrelation of the Markov chain, but also in terms of the computational cost. To be explicit, the computational cost is independent of the number of respondents, which makes our approach ideally suitable for large-scale educational measurement applications involving hundreds of thousands of respondents. The efficiency derives from our starting point, the closed form representation of the marginal Rasch model from Cressie and Holland (1983), that removes the need for any form of data augmentation. Because no assumptions need to be made regarding the distribution of ability, our approach is robust compared to other approaches that do rely on such assumptions. To wit, without assumptions there can also be no wrong assumptions, and hence no bias that may result from them. Because we in fact set up a Markov chain for the *extended* marginal Rasch model, we do not even have to assume that a distribution exists. The extended marginal Rasch model is a proper statistical model in its own right.

The alternative parametrization of the extended Rasch model in terms of the posterior expectations corresponding to the different scores ($\tau _s$) shows that the least assumption we would want to add to the model, in most educational measurement contexts, is that the sequence $\tau _s$ is non-decreasing in *s*. This assumption ensures that all the item-rest regression functions are non-decreasing, which is what we would expect from a test intended to measure a single construct. This additional assumption is easily imposed and/or tested in a Bayesian framework.

This last remark being true, it is still worthwhile not only from a theoretical, but also from a practical, point of view to keep the distinction between the proper marginal Rasch model and the extended marginal Rasch model in mind. Much of the power of latent trait models such as the marginal Rasch model derives from the fact that a complex multivariate distribution may be *reduced* to a single (latent) variable, the relation of which with all sorts of other variables (both as explained and as explanatory) is an important field of research. Keeping the distinction between the proper and extended marginal Rasch model in mind, we can have two distinct meanings.

First, we may impose on the algorithm for the extended marginal Rasch model, the proper constraints to ensure that the parameters correspond to the marginal Rasch model. The simplest approach involves imposing the inequality constraints from the reduced moment problem via the prior distribution, as we illustrated. This approach is easily implemented and only requires an efficient algorithm for sampling from a truncated beta distribution.

Second, we may want to test the fit of the proper marginal Rasch model against the extended marginal Rasch model. That is, we want to test the hypothesis $\lambda \in \Omega$ (where $\Omega$ indicates the subset of the parameter space consistent with the reduced moment problem). This takes the form of testing a set of inequality constraints. In principle, this can be accomplished using Bayes factors or via evaluating the posterior probability of $\Omega$. As this topic deserves attention in its own right, and its details extend well beyond the scope of this paper, we leave this as a topic for future research.

We perceive the use of our approach as being part of a plug-and-play divide-and-conquer approach to statistical inference for the Rasch model. The algorithm developed in this paper allows us to evaluate the fit of the marginal Rasch model, and allows for sound statistical inference on the item parameters, without the need for modelling the distribution of a latent trait. In a second step, after having concluded that the marginal Rasch model fits the data, we can start modelling the latent trait distribution. This topic will not be developed further in this paper and is also left for future research. Considering the representation of the marginal Rasch model in Eq. 6, this entails setting up a parametric model for the score distribution ($\varvec{\pi }$). Such a model is useful for the purpose of relating the latent trait to explanatory variables (e.g. for latent regression). Combining draws from the posterior distribution of the item parameters (integrating out the $\varvec{\lambda }$ parameters), with draws from the posterior distribution of population specific parameters (in a parametric family of population distributions), conditionally on the item parameters, allows for the construction of simple and robust plug-and-play algorithms for survey research.

## Footnotes

1 Where possible without introducing ambiguity, we suppress the difference between random variables and their realization.

2 The elementary symmetric function of order *s* of the vector $\mathbf {b}$ is defined as

$$\begin{aligned} \gamma _s(\mathbf {b})=\sum _{\mathbf {x} \rightarrow s} \prod _i b_i^{x_i} \end{aligned}$$

where the sum runs over all response patterns $\mathbf {x}$ that yield the sum score *s*.
